# Recent advances in structural characterization of biomacromolecules in foods *via* small-angle X-ray scattering

**DOI:** 10.3389/fnut.2022.1039762

**Published:** 2022-11-17

**Authors:** Yang Sun, Xiujuan Li, Ruixin Chen, Fei Liu, Song Wei

**Affiliations:** ^1^College of Vocational and Technical Education, Yunnan Normal University, Kunming, China; ^2^Pharmaceutical Department, The Affiliated Taian City Central Hospital of Qingdao University, Taian, China; ^3^Tumor Precise Intervention and Translational Medicine Laboratory, The Affiliated Taian City Central Hospital of Qingdao University, Taian, China

**Keywords:** SAXS, structural characterization, food biomacromolecules, modeling, *in situ* capabilities, time-resolved, chromatography, integrated structural biology

## Abstract

Small-angle X-ray scattering (SAXS) is a method for examining the solution structure, oligomeric state, conformational changes, and flexibility of biomacromolecules at a scale ranging from a few Angstroms to hundreds of nanometers. Wide time scales ranging from real time (milliseconds) to minutes can be also covered by SAXS. With many advantages, SAXS has been extensively used, it is widely used in the structural characterization of biomacromolecules in food science and technology. However, the application of SAXS in charactering the structure of food biomacromolecules has not been reviewed so far. In the current review, the principle, theoretical calculations and modeling programs are summarized, technical advances in the experimental setups and corresponding applications of *in situ* capabilities: combination of chromatography, time-resolved, temperature, pressure, flow-through are elaborated. Recent applications of SAXS for monitoring structural properties of biomacromolecules in food including protein, carbohydrate and lipid are also highlighted, and limitations and prospects for developing SAXS based on facility upgraded and artificial intelligence to study the structural properties of biomacromolecules are finally discussed. Future research should focus on extending machine time, simplifying SAXS data treatment, optimizing modeling methods in order to achieve an integrated structural biology based on SAXS as a practical tool for investigating the structure-function relationship of biomacromolecules in food industry.

## Introduction

Bioactive macromolecules, including peptides, nuclear acids, proteins, carbohydrates, and lipids, are not only essential components of food but also are the dominant substances for food to realize its functions. Meantime, biomacromolecules are widely used as additives for beverages, yogurt, cereal products, nuts, snacks, etc. to improve the food nutrition (1). Besides, other functional properties, such as gelation, foamability, water retention and emulsification, typical functions induced by structural changes of biomacromolecules, play significant roles in food processing. Therefore, the structure-function relationship of biomacromolecules is one of the most important topics in food science and technology.

Several experimental techniques are available for the structural characterization of biomacromolecules. High-resolution structural techniques, including crystallography, electron microscopy (EM) and nuclear magnetic resonance (NMR) have yielded incredibly detailed structural information at the atomic level on highly populated static states (2–4). However, due to the requirement of good crystals for crystallography, requirement of solubilized and monodisperse sample, and the low molecular weight requirement of NMR, a significant fraction of food biomacromolecules cannot be analysed using these three high-resolution methods (5). Furthermore, because of the highly heterogeneous and polydisperse of most food biomacromolecules, it is also challenging for conventional techniques, Fourier Transform Infrared spectroscopy (FTIR), dynamic light scattering (DLS) to monitor the change in the structure of biomacromolecules, especially the dynamics of self-assemble and hydrolysis. Therefore, developing of alternative structural characterization techniques for biomacromolecules with rapid response, easy sample preparation, data collection under near-native conditions, and *in situ* capability, is needed in food science and technology.

Small-angle X-ray scattering (SAXS) is a powerful tool for structural characterization of samples under resolutions ranging from a few Angstroms to hundreds of nanometers. SAXS is sensitive to both ordered and not-ordered features in the sample and it has several advantages over direct characteristic techniques in that: a very small amount of sample for measurement, rapid data collection, no crystallization or fixation requirement, high-throughput screening model and multiple *in situ* capacities, etc. (6). These features make SAXS an interesting technique for academic and industrial applications of highly interdisciplinary field, including life science, biomedicine, and biomaterial engineering.

Since SAXS was first used to study the geometry of typical milk proteins β-lactoglobulin tetramer, there have been nearly 60 years of research on food biomacromolecules. Several studies have reviewed the advances and applications of SAXS in food field (7–13). Gilbert summarized the principle and the latest activities in the application of SAXS to food colloids (14). This work provides information for the SAXS expert who is interested in applying this method to food colloids and the food scientist that wishes to gain knowledge of the former.

However, a work concluding the recent development and applications of modeling programs and *in situ* capabilities of SAXS for the structural characterization of food biomacromolecules is not available yet. Therefore, in the present review, the SAXS principle, theoretical calculations and modeling programs are summarized, technical advances in the experimental setups of *in situ* capabilities: coupled with chromatography, time-resolved, temperature, pressure, flow-through, are elaborated. Recent applications of SAXS for studying the structural properties of food biomacromolecules including proteins, carbohydrates and lipids are highlighted. Moreover, the limitations and prospects of SAXS are also discussed. We hope this review will provide reference information for food scientists who investigate the relationship between the structure and function of biomacromolecules using SAXS.

### Principle, theoretical calculation, and programs

A typical bio-SAXS measurement is performed using a sample concentration at least ∼0.5–10 mg ml^–1^ with a ∼15–30 μl of volume, and generally takes less than a few minutes on a synchrotron beamline or dozens of minutes to hours using an in-house instrument (15). The principle of SAXS is that a collision between a monochromatic incident X-ray beam and a surface particle results in scattering of the beam in all directions. The one-dimensional (1D) scattered intensity *I*(*q*) and the average of the various conformers present in the population of scattered particles are recorded using a two-dimensional (2D) detector. The magnitude of the scattering vector *q* = 4πsinθ/λ, where θ is the half of the angle between incident and scattered beams. At small angles (θ < 5°), the inhomogeneity in the electron clouds can be observed, which will provide information about the size and shape of biomacromolecules in the sample (16). The “background” scattering from the buffer is independently measured and subtracted from that of the solution (17).

**FIGURE 1:**
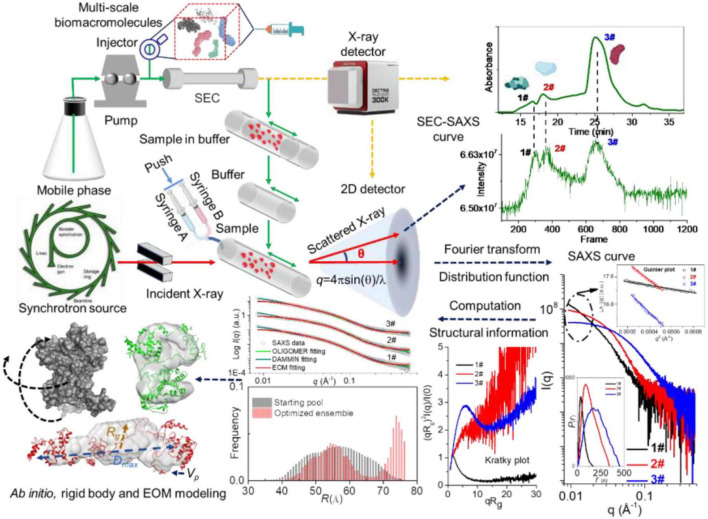
Principle, a typical small-angle X-ray scattering (SAXS) experiment and derivative profiles. Reproduced from Sun et al. (29) (Copyright 2021 Elsevier Publisher).

The radius of gyration (*R*_*g*_) of biomacromolecules can be estimated directly from small *q* values using Guinier approximation (18), $I\,\left(q\right)=I\,\left(0\right)\,\text{exp}\,\left(\frac{-q^{2}\,R_{g}^{2}}{3}\right)$, where *I*(*q*) is the scattering intensity and *I*(*0*) is the forward scattering intensity. The pair-distance distribution function *p*(*r*) (19), corresponding to the paired set of distances between all electrons within the scattered particle, can be generated *via* indirect Fourier transform by using the GNOM (20), PRIMUS (21), BioXTAS RAW (22), and BIFT programs (23). By comparing with molecular weight determined from *I*(0), the volume of the biomacromolecules can be calculated by the Porod approximation (24, 25). Moreover, the compactness or flexibility of biomacromolecules can be evaluated using the Kratky (26), dimensionless Kratky (27), and Porod-Debye plots (28). The principle, a typical SAXS measurement and derivative profiles can been seen from Figure 1.

**FIGURE 2:**
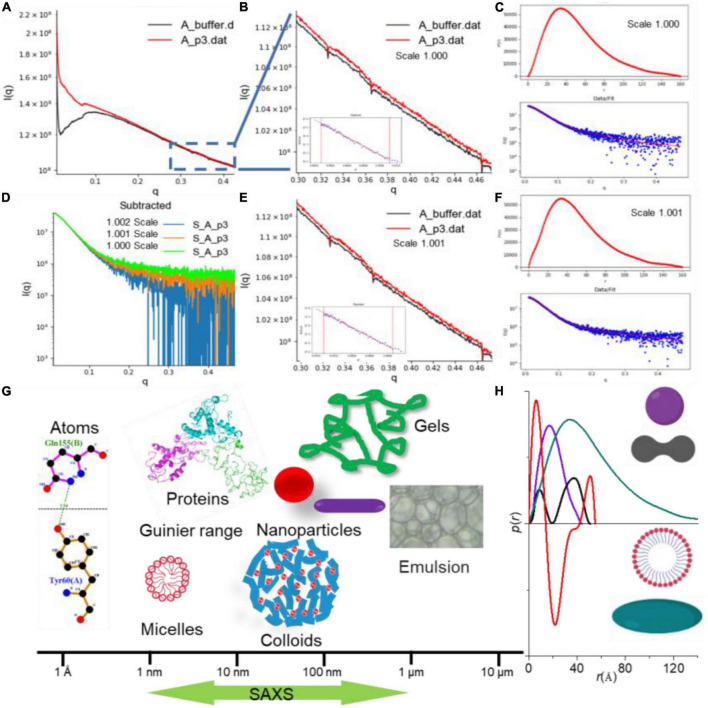
The small-angle X-ray scattering (SAXS) data analysis by RAW program (22). **(A)** Background subtraction of caseinate (29). **(B–F)** Scattering curves showing changes in net scattering intensity in the high *q* region with different scale factors (1.000 and 1.001), as well as the resulting *p*(*r*) in different scaling factor. **(G)** Size determination coverage of SAXS. **(H)** *p*(*r*) profile depends on the dimension and shape of the biomacromolecule.

The net scattering intensity is critically dependent on the scale factor, especially at high *q* regions (as shown in Figure 2A), and any minor deviation from the accurate value may have a significant impact on the final results (30). As seen from Figures 2B–F, a sharp decrease in the net scattering intensity at high *q* regions is observed due to a slight increase in scale factor (1.000–1.001) for background buffer subtraction. SAXS allows the determination of the structural properties under a broad range from a few Angstroms to hundreds of nanometers, which covers the size of biomacromolecules and their complex (Figure 2G). The maximum distance within a scattered particle (*D*_*max*_) can be assessed by the distance *r* of the *p*(*r*) distribution equals zero (Figure 2H). When the *p*(*r*) curve with a maximum frequency at a distance less than half of the *D*_*max*_ (olive curve in Figure 2H), the scattered particle adopts rather extended and elongated conformation in solution. Therefore, the change in conformation and structure of biomacromolecules can be monitored by *p*(*r*) profile. Moreover, an accurate determination of the scale factor for background subtraction has significant implications for obtaining the further reliable structural parameters of biomacromolecules (25).

Small-angle X-ray scattering profile can then be used for a further series of theoretical calculations to obtain structural information, *ab initial* model and ensemble structures. By translating scattering curves into bead representations consisting of a set of dummy atoms, an *ab initio* model of biomacromolecule with a resolution of 10–30 Å can be obtained *via* DAMMIN (31) and GASBOR programs (32). It is noted that DAMMIN is the most used in the modeling programs for reconstructing low-resolution shape of biomacromolecule. An iterative phase retrieval method, density from solution scattering (DENSS), has been expanded to analyze SAXS data, which may avoid many of the assumptions limiting the resolution and accuracy of modeling algorithms by explicitly calculating electron density (33). Deep learning methods, such as DecodeSAXS, have been reported used to classify and reconstruct the shapes with model parameters (34, 35). Critical Assessment of Protein Structure Prediction (CASP), a machine learning program, utilizes SAXS data to build improved models simulating the global shape of the target (36). Google’s AlphaFold 2 indisputably won the CASP14 competition. The results are so incredibly accurate that many have hailed this code as the solution to the long-standing protein structure prediction problem.

MONSA program (31) can be used to treat biomacromolecules containing multiple phases (e.g., intensities from protein/nucleic acid complexes) (6). SREFLEX (37), SASREF (38), and CORAL (39) programs can be used for high-resolution modeling of rigid bodies. SAXSDom has been utilized to model stable multi-domain proteins with flexible linker regions (40). Moreover, CRYSOL is used to compare the SAXS data with a PDB file with an X-ray or NMR structure of a protein or a protein-DNA(RNA) complex (41). SUPCOMB is a tool for superimposing one 3D structure onto another (42). In the case of flexible and mixed biomacromolecule systems (protein, DNA, and RNA), the structural ensembles of the biomacromolecule can be acquired by the ensemble optimization method (EOM) (43). For the oligomeric and transient state system, the volume fraction of components can be calculated using OLIGOMER, and the *ab initio* model of intermediate may be acquired by DAMMIX (21, 44). A hybrid resolution molecular dynamic (MD) method (hySAXS) has been used to create an ensemble of structures for intrinsically disordered proteins (IDPs), which is comparable to the experimental SAXS data (45). The extended experimental inferential structure determination method (X-EISD) (46) and Bayesian/Maximum entropy (BME) method (47) can be also used to calculate the maximum log-likelihood of an IDPs ensemble derived from SAXS. LIPMIX (48) and BILMIX programs (49) enable polydispersity of the model in multilamellar and asymmetric lipid vesicles and simultaneously generate the corresponding size distribution, respectively. The program ELLLIP can reconstruct the quasi-atomistic models of ellipsoidal liposomes (50) as well as bi-micellar systems involving proteins (51).

Several integrated docking methods by fitting the theoretical scattering curve of possible models to the experimental SAXS data have been developed to estimate the structure of complexes. Examples of such docking methods include SASREF (38), FoXSDock (52), HADDOCK (53), ClusPro FMFT-SAXS (54), pyDockSAXS (55), RosettaDock_*SAXS*_ (56), PatchDock (57), and ATTRACT-SAXS (58). It has shown that iSPOT can filter docked structure and characterize a native-like model combined SAXS with foot-printing data by generating theoretical scattering of crystal structure (59). Molodenskiy et al. presented a PyMOL plugin, MPBuilder, which provided a set of adaptable routines for modeling membrane proteins (MPs), protein-detergent complex, bicelles, and lipid scaffold (saponin nanoparticles, nanodiscs) validated with SEC-SAXS data (60).

A comprehensive list of programs to reconstruct the structure and model of biomacromolecules based on SAXS data is shown in Table 1, and many of the programs are publicly available to academic users and moderately easy to operate. The details and applications of each program please see the corresponding reference. The popularity of SAXS has been propelled by novel data analysis and modeling algorithms. Developing user-friendly modeling programs will facilitate the utilization of SAXS for large-scale studies, which is also a major achievement in the community toward broader use of the method in combination with complementary techniques and enabling the cross-validation of structural data (61).

**TABLE 1:** List of some of the available software programs used in the analysis and reconstruction of models based on small-angle X-ray scattering (SAXS) data.

Program	Accepted experimental file	Functionality	Output	Web server	References
Membrane protein (MP) Builder	The plugin of both PyMOL and ATSAS, SAXS data	Generation and refinement of all-atom protein-detergent, bicelle, and lipid-scaffold (saponin nanoparticles, nano-discs) complexes	Models of protein-detergent assembles without minimized energy	https://github.com/emblsaxs/MPBuilder	(60)
Critical assessment of protein Structure prediction (CASP)	SAXS data	SAXS-assisted protein structure prediction	Predicted solution structure	https://predictioncenter.org/	(36)
CRYSOL in ATSAS	PDB, SAXS data	Evaluating atomic structure of biomacromolecules based on SAXS experimental data	Fitting with chi values	https://www.embl-hamburg.de/biosaxs/crysol.html	(51)
CORAL combines the algorithms of SASREF, BUNCH in ATSAS	PDB, SAXS data	Rigid body modeling of multidomain protein complexes against multiple SAXS data	PDB and fitting	https://www.embl-hamburg.de/biosaxs/manuals/coral.html	(39)
DAMMIN or MONSA in ATSAS	Output file of the program GNOM in ATSAS	Restoring *ab initio* shape of biomacromolecules	PDB and fitting	https://www.embl-hamburg.de/biosaxs/manuals/dammin.html	(31)
GASBOR in ATSAS	Output file of the program GNOM in ATSAS	Restoring *ab initio* of protein structure using a chain-like ensemble of *dummy residues*	PDB-alike file	https://www.embl-hamburg.de/biosaxs/manuals/gasbor.html	(32)
OLIGOMER in ATSAS	PDB, SAXS data	Computation of volume fractions of mixtures of protein with SAXS data from the components	Fitting and file containing volume fractions of components in mixture	https://www.embl-hamburg.de/biosaxs/manuals/oligomer.html	(21)
DAMMIX in ATSAS	PDB, SAXS data	Restoring *ab initio* shape of intermediate state component and its volume fraction	PDB and fitting	https://www.embl-hamburg.de/biosaxs/manuals/dammix.html	(44)
EOM in ATSAS	Amino acid sequence, PDB of domains/subunits, SAXS data	Fits an average theoretical scattering intensity derived from an ensemble of conformations to experimental SAXS data.	PDB and fitting	https://www.embl-hamburg.de/biosaxs/manuals/eom.html	(43)
FoXS	PDB, SAXS data	Computing a theoretical scattering profile of a structure and fitting of experimental profile	Fitting file of PDB with SAXS curve	https://modbase.compbio.ucsf.edu/foxs/	(52)
SAXSDom	Sequence of individual domain	Multidomain protein assembly modeling	PDB file of multidomain protein	https://github.com/jianlin-cheng/SAXSDom	(40)
FoXSDock	PDB files of receptor and ligand, SAXS data	Docking two rigid protein structures based on a SAXS profile of their complex	PDB file of complex	https://modbase.compbio.ucsf.edu/foxsdock	(52)
ATTRACT-SAXS	PDB files of receptor and ligand, SAXS data	Docking protein-protein benchmark with simulated SAXS data without a physiochemical force field	High-quality solution models of protein-protein complexes.	http://www.attract.ph.tum.de/services/ATTRACT/attract.htmltum.de	(58)
RosettaDockSAXS	SAXS data	Predicting unknown 3D atomic structures of protein-protein complexes	3D atomic structures	https://rosie.rosettacommons.org/docking/	(56)
DecodeSAXS	SAXS data	Machine learning methods to build 3D models	3D models	http://liulab.csrc.ac.cn:10005/submit/	(35)
pyDockSAXS	PDB files of receptor and ligand, SAXS data Complex type: enzyme inhibitor, antibody, or antigen	Structural models of protein–protein interactions at large scale.	Models of complex	life.bsc.es/pid/pydocksaxs	(114)
ClusPro	PDB files of receptor and ligand	Protein–protein docking server based on fast Fourier transform (FFT) data	Models of complex	http://cluspro.org/nousername.php	(115)
X-EISD	Sequence of protein, SAXS experimental data	Generating ensembles of IDPs	Ensembles	https://github.com/THGLab/X-EISD	(46)
BME	Experimental data. Calculated data from simulation trajectory	Generating ensembles	Ensembles	https://github.com/KULL-Centre/BME	(47)
SAXScreen	SAXS data, ITC titration curve, ligand, and buffer SAXS data	Screening protocol utilizing SAXS to obtain structural information involving protein-RNA interactions.	Models of complex	https://github.com/zharmad/SAXScreen	(116)

## *In situ* capabilities of small-angle X-ray scattering

### Size-exclusion chromatography-small-angle X-ray scattering

The online purification system coupled with SAXS, such as size-exclusion chromatography (SEC), gel filtration chromatography, and reversed-phase chromatography, is a standard approach for separating oligomeric species or components in a heterogeneous sample (62). The programs like CHROMIXS (63), DATASW (64), DELA (65), EFAMIX (66), and US-SOMO HPLC-SAXS module (67) have been developed to process chromatography-SAXS data. The scheme for SEC-SAXS setup is shown in Figures 1, 6, and applications of chromatography combined with SAXS for studying biomacromolecules are summarized in Table 2.

**TABLE 2:** Application of chromatography combined small-angle X-ray scattering (SAXS) for studying biomacromolecules.

Samples	X-ray source	Elution buffer	column	Concentration/ volume	Detector distance from sample	Flow	Exposure	References
BSA	EMBL P12 beamline	50 mM HEPES, 150 mM NaCl, 2% v/v glycerol, pH 7	Superdex 200 Increase 10/300	8.8 mg ml^–1^ 100 μl	Pilatus 6M, 3.3 m	0.5 mL min^–1^ + splitter	1 s	(117)
Glucose isomerase, *Streptomyces rubiginosus*	EMBL P12 beamline	50 mM Tris, 100 mM NaCl, 1 mM MgCl_2_, 1% v/v glycerol, pH 7.5	Superdex 200 Increase 10/300	10.3 mg ml^–1^ 100 μl	Pilatus 6M, 1.5 m	0.5 ml min^–1^ + splitter	1 s	(117)
Class II pyruvate aldolase	EMBL P12 beamline	20 mM HEPES, pH 7.5	Superdex 200 Increase 10/300	8 mg ml^–1^ 80 μl	Pilatus 6M, 3.3 m	0.5 ml min^–1^ + splitter	1 s	SASDEX9
Ovalbumin + β Amylase mixture, Ovalbumin	EMBL P12 beamline	20 mM Tris, 150 mM NaCl, 5% glycerol	Superdex 200 Increase 10/300	15 mg ml^–1^ 100 μl	Pilatus 2M, 3.0 m	0.5 ml min^–1^ + splitter	1 s	(118)
Cycloamylose	SPring-8	Milli-Q water or 6% (v/v) methanol	30 × 1000 mm packed with TOSOH HW-55S	2.0 mg ml^–1^ 2.0 ml	Hamamatsu Photonics V5445P-MOD, 1.5 m	2 ml min^–1^	2 s	(119)
Caseinate	SSRF BL19U2 beamline	10 mM Tris-HCl pH 6.7	Superdex 200 Increase 10/300	25 mg ml^–1^ 150 μl	Pilatus 1M, 3.0 m	0.5 ml min^–1^	1 s	(29)
Ovalbumin Proteoglycans	KEK BL-10C station	10 mM PBS, pH 6.9 50 mM PBS, pH 6.9.	SB-806 M HQ, 300 × 8 mm, Shodex GF-7M HQ, Shodex	0.735 mg ml^–1^ 3% (w/v)	Pilatus3 2M, 1.98 m	0.3 ml min^–1^ 0.4 ml min^–1^	3 min with intervals of 10 s	(120, 121)
Glucose isomerase	Australian Synchrotron coflow-SEC SAXS beamline	20 mM PBS with 128 mM NaCl, 22 mM KCl, 5% (v/v) glycerol, pH 7.5	Superdex S200 Increase 5/150	2.5 mg ml^–1^ 100 μl	Pilatus2 1M, 8 m	0.5 ml min^–1^	2 s	(122)
Lysozyme	PLS II 4C SAXS beamline	10 mM PBS with 138 mM NaCl, pH 7.4	Agilent Bio SEC-5	20 mg ml^–1^ 100 μl	Rayonix 2D, no mention	0.06 ml min^–1^	10 s	(123)
Immunoglobulin G	La-SSS adopts NANOPIX	100 mM Tris-HCl with 100 mM NaCl, pH 7.5	Superdex 200 Increase 10/300	5.0 mg ml^–1^ 500 μl	HyPix-6000, 0.35 m	0.02 ml min^–1^	30 s	(124)
Apoferritin	BioXolver L, Xenonx	50 mM HEPES, pH 7.5	Superdex 200 Increase 10/300	0.5 mg mL^–1^ 500 μL	Single-photon-counting detector, 0.6 m	0.5 ml min^–1^	30 s	(125)
Yeast alcohol dehydrogenase	EMBL P12 beamline	50 mM HEPES, 150 mM NaCl, 2% v/v glycerol, pH 7	Superdex 200 Increase 10/300	9.2 mg ml^–1^ 100 μl	Pilatus 6M, 3 m	0.5 ml min^–1^	67 s	(117)

Although the nanocluster model for describing casein micelle structure is widely accepted, little direct evidence at the nanometer scale supported this model. Sun et al. (29) reported a method that can prove and quantify the conformation and the fine structure of the casein cluster based on SEC-SAXS. The SEC-SAXS results showed that casein cluster presented *R*_*g*_ values ranging from 39.45 to 40.77 Å with a *D*_*max*_ of 180 Å. The dimensionless Kratky plot suggested a rather extended and elongated conformation of casein cluster in solution. The experimental *M*_*w*_ according to the Bayesian Interference analysis was 50.3–64.7 kD with a probability of 91.54%, indicating the presence of 2–3 casein monomers in the cluster. Further, the DAMMIX and OLIGOMER results indicated that the cluster consisted of four species, α_*s1*_-β-α_*s2*_-casein, α_*s1*_-casein, α_*s2*_-casein and α_*s1*_-α_*s2*_-casein with a volume fraction of 64.3, 22.8, 8.5, and 4.4 %, respectively. The results of EOM indicated the presence of two conformers in α_*s1*_-β-α_*s2*_-casein, the elongated one (∼60 Å of *R*_*g*_) with 64.7% of volume fraction and the compact one (∼35 Å of *R*_*g*_) with 35.3% of volume fraction. It is the first time to reveal the structural properties of casein cluster based on SEC-SAXS, which may help understand better for internal structure of casein micelles regarding their primary casein cluster. Therefore, SAXS has been proved to be a powerful tool to study the structure and dynamics of the flexible, disordered and mixed biomacromolecules.

### Time-resolved and time-dependent small-angle X-ray scattering

Small-angle X-ray scattering measurements are performed over a set time period ranging from microseconds to hours to assess the time-resolved (TR-SAXS) and time-dependent (*in situ* or real time) changes in structure and function for protein, carbohydrate, fat or non-nutritive compounds, such as gelatinization, assembly, micellization or colloid formation as well as in digestion and hydrolysis (68). This method is particularly suited to differentiate triggers of structural changes, including optical excitation (69), electron transfer (70), temperature jump (T-jump) (71), pH-jump (72), photoreduction (73), and reactant concentration jump (74).

**FIGURE 3:**
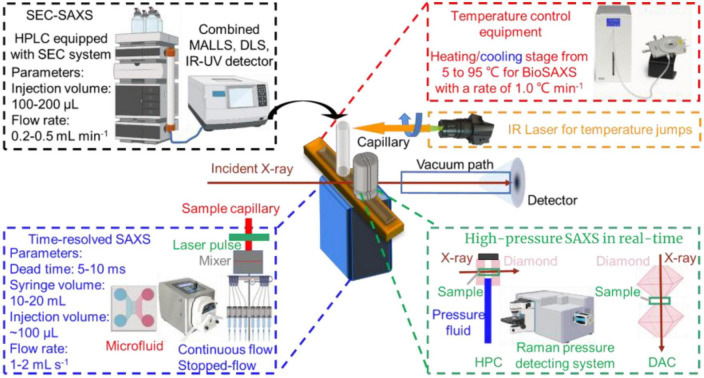
*In situ* capabilities of small-angle X-ray scattering (SAXS).

Kuang et al. investigated the lamellar structure change of waxy corn starch during gelatinization and reveal the gelatinization mechanism by TR-SAXS in the temperature range from 35 to 141.85 °C with a measurement of 60 s at each degree (75). Gilbert reviewed the latest activities in the application of time-dependent SAXS to food colloids (14). Hempt et al. reported a novel digestion model of milk using an integrated online flow-through TR-SAXS with an *in vitro* cell co-culture model (76). Krishnamoorthy et al. reported an approach based on the time-dependent SAXS from protein spherical nucleic acids to elucidate the enzymatic degradation of DNA, which should prove invaluable in probing other enzyme-catalyzed reactions on the nanoscale (77). The details of TR-SAXS equipped with the laser pulse recording as a function of the time delay between laser pulse and incident X-ray are shown in Figure 3.

The SAXS beam equipped with the microfluidic device (continuous flow and stopped-flow) may not only reduce the sample damage by radiation (78), but also monitor the dynamic structural alternations during interactions in real-time (79). Hsu et al. characterized the transient partially folded state of bovine α-lactalbumin (BLA) coupled with TR-SAXS following a T-jump (74). The structural responses of BLA after an 11.5°C T-jump from the initial temperatures of 60, 65, and 70°C were individually recorded from 20 μs to 70 ms delay. Three states were molten globule state and two terminal unfolded states, U_1_ and U_2_. The application of TR-SAXS for structural characterization of biomacromolecules in foods is listed in Table 3.

**TABLE 3:** Application of time-resolved small-angle X-ray scattering (TR-SAXS) at various synchrotron beamlines.

Samples	X-ray source	*q*-Range	Acquisition time	References
Gluten protein mixtures	ESRF beamline ID02	1.2 × 10^–4^–6.0 × 10^–3^ Å^–1^	5 ms	(126)
Gelation of pea and whey proteins	APS beamline 9-ID-C	1.0 × 10^–4^–0.3 Å^–1^	A 90 s measurement every 2–5 min	(127)
Polyphenol pea protein gel	APS beamline 9-ID-C	1.0 × 10^–4^–1.0 Å^–1^	20 s	(128)
Zein-based oleo gel	APS beamline 9-ID-C	1.0 × 10^–4^–1.2 Å^–1^	20 s	(129)
Liquid–liquid phase separation of BSA-YCl_3_ system	ESRF beamline ID02	9.0 × 10^–5^–7.0 × 10^–3^ Å^–1^	5–50 ms	(130)
Cellulose nanofibers	NSLS beamline X9	1.0 × 10^–3^–0.3 Å^–1^	10 s	(131)
Gelation of amylose	SPring-8 BL-40B2	1.0 × 10^–4^–0.8 Å^–1^	1–62 min	(132)
Lipid/surfactant assemblies	ESRF beamline ID02	3.0 × 10^–3^–0.19 Å^–1^	20 ms	(133)
Waxy corn starch	SSRF BL16B1	0.025–0.15 Å^–1^	60 s	(75)
Milk lipid crystallization during digestion	Australian Synchrotron SAXS beamline	0.005 < *q <* 1.0 Å^–1^	5 s	(68)
Milk digestion in presence of a cell	Swiss Light Source SAXS beamline	0.006 < *q <* 0.5 Å^–1^	70 min with an exposure time of 1 s and a 9 s delay	(76)
Krill oil-in-water emulsion	EMBL P12 beamline	0.01 < *q <* 0.5 Å^–1^	3,500 s with a 1 s exposure and 9 s delay	(108)
Oleic acid (OA) and glycerol monooleate (GMO) self-assemblies	ELETTRA Austrian SAXS beamline	0.018 < *q <* 0.5 Å^–1^	Five frames with an exposure time of 20 s	(134)
Liquid depot formulations	ELETTRA Austrian SAXS beamline	0.02 < *q <* 0.5 Å^–1^	10 min with a 5 s exposure with 5 s delay	(135)
Lipid vesicles and Ca^2+^	ESRF beamline ID02	0.0067 < *q <* 0.5124 Å^–1^	35 frames, first frame 0.04 s after mixing, last frame 316.16 s with a 0.02 s exposure	(136)
Soy phosphatidylcholine-citrem nanoparticles	ELETTRA Austrian SAXS beamline	0.01 < *q <* 0.4 Å^–1^	Four frames with a 0.25 s exposure	(137)

### Temperature

Temperature is one of the most important parameters controlling the formation, morphology, and structure of biomacromolecules, since much of biochemistry is thermally driven, functionally relevant conformational changes can also be triggered by changes in temperature. Generally, a trigger T-jump using a nanosecond laser pulse or an infrared (IR) light with a wavelength of 1450 nm (Figure 3, right upper) can be applied to most temperature-sensitive biomacromolecules to perturb the structural dynamics and reveal the changes in structural kinetics and association under various temperature conditions (80).

Berntsson et al. developed a CoSAXS beamline for millisecond T-jump experiments tracked by TR-SAXS with the Eiger2 and Mythen2 detectors and recorded the scattering of the solvent (80). A ∼15°C T-jump can be triggered by a 2 ms infrared laser light and maintained for several seconds with additional laser pulses. The structural changes in lysozyme induced by a T-jump were observed and the population of lysozyme structures differed at this temperature. Moreover, the data showed that IR radiation absorbed directly by the solvent did not show a significant effect compared with that induced by the thermal changes in the protein. Thus, the temperature induced change in structure of biomacromolecule and thermal dynamics of system can be monitored by SAXS effectively based on the scattering characteristic of sample in SAXS profile.

### Pressure

High-pressure (HP) food treatment including pasteurization, sterilization, and shelf-life extension, has widely been used to ensure food safety and preserve various thermally sensitive nutrients and bioactive compounds (81). HP-SAXS can also be used to track a wide range of structural changes of food biomacromolecules under pressure in real time (82). Moreover, the experimental setup of TR-SAXS studies of kinetic events induced by sub-millisecond timescale hydrostatic pressure jumps (P-jump, 1–5,000 bar) is also available in several synchrotron SAXS beamlines (83). Typically, the diamond anvil cell (DAC) covers the measurement of milk, solid powders, crystals and crystalline liquids (84). Hydrostatic pressure cell (HPC) is widely used to study phase diagrams of lipid, nano-assemblies, or pressure-dependent structure-function of biomacromolecules (85, 86).

Lehmkühler et al. reported the pressure-induced formation of super crystals from high-quality PEGylated colloidal nanoparticles using 5 ms P-jump SAXS (87). They demonstrated the crystallization pressure (*p*_*c*_) of the suspension by tracking SAXS patterns at pressures above 2 kbar in steps of 100 bar and verified *p*_*c*_ between 2.9 and 3 kbar. They observed that the pressure (*p*_*f*_) jumped from 2.9 to 3.58 kbar averaging over 200 ms exposure time. The characteristic time (tw) decreased from 6.1 to 0.07 s with a reduction in Bragg reflection width from 0.138 to 0.0458 Å^–1^, suggesting the higher the *p*_*f*_, the faster the formation of nanoparticle structure. The results showed that a larger P-jump induced attractive interactions and thereby accelerated the formation of colloidal nanocrystal superlattices with enhanced crystal quality. Therefore, HP-SAXS can be utilized to track the structural change during interactions of biomacromolecules as well as to monitor the preparation of various biobased nanostructures. Exploiting easy operation setup will broad the applications of SAXS for complex biomacromolecule system.

## Applications of small-angle X-ray scattering in characterizing food biomacromolecules

### Proteins

As one of the most significant biomacromolecules in food, protein plays an essentially nutritional role *in vivo*. Meanwhile, protein-based ingredients fulfill several technical functions in food formulations and contribute to texture, color, flavor, and other properties such as solubility, stability emulsification, gelation and foaming (88). These researches involve studying protein structure-function relationships, optimizing the utilization of the components of the product, improving the quality, reducing costs, and developing novel protein application (89). SAXS is one of the most suitable techniques for protein structure and function relations study.

According to Yang et al. SAXS was used to investigate the nanostructure of quinoa protein (*Chenopodium quinoa*) isolates (QPI), one of the emerging proteins native to South America with a well-balanced amino acid profile, and the effect of NaCl and CaCl_2_ on the heat-induced gelation of QPI (90). Thermal treatment increased the sample *I*(*q*) in low-*q* region and the scattering intensity remained almost the same in the high-*q* region, which suggested that heat-induced QPI aggregation and then gelation merely occurred on the micron scale, while the internal structure of QPI on the nanoscale changed little. A Guinier shoulder in the mid-*q* region (0.02 Å^–1^< *q* < 0.08 Å^–1^) of the Kratky plot suggested the existence of nanoscale protein particles or inhomogeneities in QPI gel containing 0–200 mM NaCl. By fitting with correlation length model ($\text{I}\,\left(\text{q}\right)=\frac{\text{A}}{{\text{Q}}^{\text{n}}}+\frac{\text{C}}{{\left(\text{Q}\text{ζ}\right)}^{\text{m}}}$), the correlation length (ζ) or particle size of ∼32 Å was obtained for all the QPI gels containing 0–200 mM NaCl. Calcium binding or protein cross-linking induced minor protein inhomogeneities as indicated by substantial changes in the SAXS curve as well as a small peak at *q*∼0.2 Å^–1^ in SAXS patterns of QPI gel containing CaCl_2_.

**FIGURE 4:**
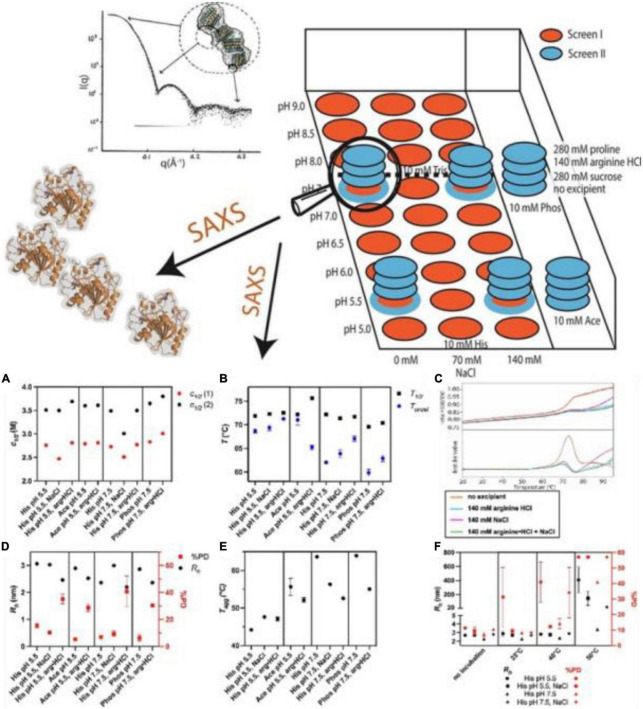
**(Top)** Combination of high throughput and structural screening to assess protein stability using small-angle X-ray scattering (SAXS). **(Bottom) (A–F)** High throughput stability screening of *Thermomyces lanuginosus* (TLL) (91) (Copyright 2022 Elsevier Publisher).

Pohl et al. (91) reported a high-throughput SAXS screening approach to assess the conformational stability and initial dispersion state of *Thermomyces lanuginosus* (TLL) and *Rhizomucor miehei* (RML), important lipases used in the food industry. They found repulsion in nine different kinds of the buffer as indicated by the decreased intensity in the low *q*-region induced by interparticle diffraction, and a significantly reduced repulsion and reduced oligomerization in phosphate buffer. Salt (35, 70, 140 mM NaCl) had minimal impact on SAXS profiles of TLL in histidine buffer at pH 5.5 and pH 7.5. The major species in the solution in all conditions was found to be monomeric, which confirmed that the differences in SAXS data were related to protein-protein interaction, suggesting that SAXS is used more widely as a tool to gain in-depth knowledge especially for the later stages of protein formulation in the food industry (Figure 4).

**FIGURE 5:**
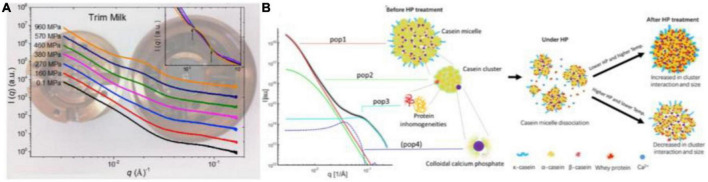
**(A)** *In situ* synchrotron small-angle X-ray scattering (SAXS) patterns of trim milk under different HHP using DAC (92) (Copyright 2018 Elsevier Publisher). **(B)** Graphic representation of the four contributions of CNs model at different length scales and structural dynamics during HHP from *in situ* SAXS, based on Yang et al. (84) (Copyright 2021 Elsevier Publisher).

The structure of casein micelles contributes to the primary physicochemical and organoleptic properties of milk. Yang et al. (92) analyzed the changes in the internal structure of CNs under HHP (up to ∼1,000 MPa) using *in situ* HP-SAXS equipped with DAC at room temperature. They found a decrease in both scattering intensities at low *q* (∼0.003 Å^–1^) and high *q* (∼0.08 Å^–1^), suggesting the disruption of CNs and solubilization of the colloidal calcium phosphate (CCP) nanoclusters under HP treatment. The SAXS profiles under pressures ranging from 270 to 960 MPa showed two isosbestic points at *q* values of ∼0.013 and 0.03 Å^–1^, which confirmed the appearance of “sub-micelles” and dissociation of CCP. When the pressure returned to atmospheric pressure, the CNs structure reverted partially to the native one (Figure 5A). Similarly, Yang et al. (84) reported the hierarchical structure of milk at various lengths under a pressure of 200 or 400 MPa at 25, 40, or 60°C using HP-SAXS (Figure 5B). The changes in CNs nanostructures varied with pressure rather than time, and temperature played a central role during the HP process.

**FIGURE 6:**
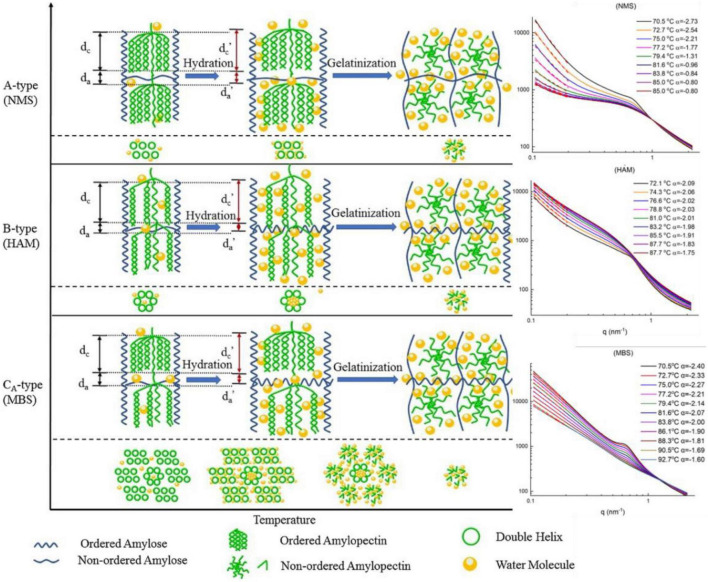
**(Left)** Schematic representation of lamellar and crystalline structural dynamics during heating. The two steps are shown: hydration, which is the uptake of water and swelling of starch granules; gelatinization, which is the disruption of starch crystal structure. **(Right)** Small-angle X-ray scattering (SAXS) profiles of HAM, NMS, MBS (95) (Copyright 2020 Elsevier Publisher).

Consequently, SAXS can be used as an effective technique not only to track the dynamic properties of biomacromolecule interactions but also to monitor the internal structure of biomacromolecule assembles.

### Carbohydrates

Carbohydrates contribute to the bulk of dietary energy and play a vital role due to their diverse biological properties and functionalities in the food industry, as a thickening, gelating, emulsifying, encapsulating, or bulking agent (93). Starch, as an important polysaccharide macronutrient, determines the processing and nutritional quality of starch-based foods (94). Increased attention is needed to identify significant opportunities for real-time monitoring of structural changes during starch processing, such as swelling, gelatinization, retrogradation, and digestibility of starch.

Liu group reported dynamic changes in lamellar structure and gelatinization of cereal starches with different amylose contents in real time using *in situ* SAXS (Figure 6; 75, 95, 96). In the low-*q* region, the curves fitted with a simple power law equation, *I*(*q*)∼*q*^–^*^α^*, where mass fractal dimension (0 < α < 3) was an indication of compactness, whereas the surface fractal dimension (3 < α < 4) was considered smooth. During gelatinization, all cereal starches showed a decreasing α value in the *q*-region between 0.01 and 0.02 Å^–1^, with the corresponding size of ∼30 to ∼60 nm with the temperature increasing from ∼70 to ∼90°C, implying a mass fractal structure of the starch gel. Interestingly, an isosbestic point in the middle *q*-region was observed for all samples, which confirmed a two-step gelatinization of starches, namely, two-correlation length (ξ) of particles in the paste/gel system. The structural paraments of lamellae, the average thickness of amorphous layers (*d*_*a*_), crystalline and amorphous layer thickness (*d*_*c*_) and the long period distance (*d*_*ac*_) parameters were calculated based on Lorentz-corrected SAXS profiles. For high-amylose maize starch (HAM), *d*_*c*_ increased from 65°C with a decreased *d*_*a*_ value, demonstrating swelling of the lamellae following water uptake. For normal maize starch (NMS) and mung bean starch (MBS), *d*_*c*_ increased from 60°C with a decreasing value of *d*_*a*_. Both *d*_*ac*_ and *d*_*c*_ rapidly decreased at 72.2, 70.2, and 69.4°C for the high amylopectin (HAP), normal rice starch (NS) and HAM samples, respectively (96).

Starch has a strong tendency to retrograde and undergoes syneresis on cooling, namely, retrogradation. The retrogradation starts with the self-assembly of amylose to form a double helix during the cooling and storage of starch gel, followed by the partial crystallization of branched polymers (amylopectin) after prolonged storage (97). Zeng et al. (94) reported the SAXS patterns of retrograde starch with α values of all samples ranging from 1.32 to 2.43, indicating the mass fractal structures of all retrograde starch samples. Compared with storage day 1, the fractal dimensions (*D*_*m*_) on storage day 24 increased from 1.32 to 2.30, which was consistent with the formation of ordered crystalline structures in the long-range and an increase in the ordered structure of starch during storage.

Starch digestibility based on sustained dietary energy and low glycemic index (GI) of foods plays a vital role in public health (98). Yang et al. used SAXS to investigate thermally and enzymatically digested corn starches under various treatment times. The semi-crystalline lamellar structure of starch exhibited a scattering peak at a *q*-region of 0.06–0.07Å^–1^ with a size of 9-10 nm corresponding to the alternating crystalline and amorphous lamellar structure of amylopectin. The peak area of thermally treated and enzymatic digested starch was quantified by fitting SAXS data (0.02Å^–1^ < *q* < 0.2Å ^–1^) with a power-law function combined with a Lorentzian peak with $I\,\left(q\right)=B+C\,q^{-\alpha }-\frac{2\,A}{\pi }\,\left(\frac{W}{4\,{\left(q-q_{0}\right)}^{2}+W^{2}}\right)$ (99). The thermal treatment induced water uptake in the amorphous regions of the granule during heating, leading to an increase in the intensity of the low *q*-region. In contrast, enzymatically treated samples showed changes in the crystalline and amorphous regions within the semi-crystalline lamellar structure and the amorphous growth rings. However, both treatments had little impact on the mass fractal structures of starch as the power law exponent (*P*) and long period distance (*d*) were around ∼2 and ∼10 nm for all samples.

Overall, the structural parameters of biomacromolecules derived from SAXS data facilitated the determination of the structure-function relationship and the evolution of nanomaterials based on the role of carbohydrates in the food industry.

### Lipids

Fats and oils are important sources of energy and nutrition, and contribute to the desirable functionality, texture and palatability of foods (100). Chemically, fats consist mainly of triglycerides (TAGs) combined with free fatty acid moieties. Besides nutritional properties, lipids facilitate the delivery of lipophilic nutraceuticals. Lipid-based colloid formation or oleogelation is designed to simulate the structure and semi-solid rheological behavior, and is widely utilized in the food industry, including coating, bakery, dairy products, meat, plant-based and artificial meat products (101).

**FIGURE 7:**
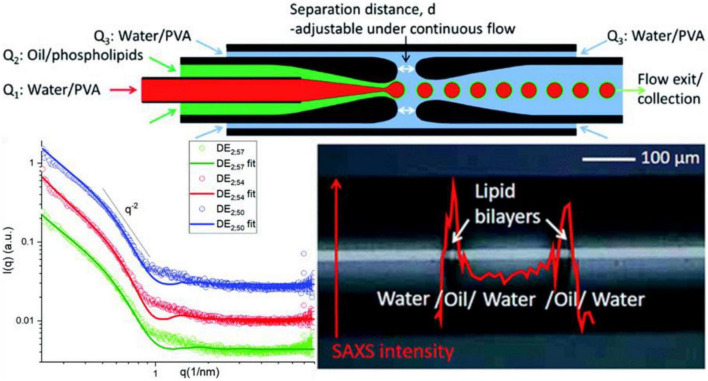
**(Top)** Setup of the microfluidic small-angle X-ray scattering (SAXS) device. **(Bottom left)** SAXS profiles of the w/o/w double emulsion interfaces obtained using the microfluidic device (RT; green), after storage at 50°C for 120 min (red) and for 240 min (blue), respectively. **(Bottom right)** Scheme of SAXS intensity at *q* = 0.9 nm^– 1^ recorded along an axial line covering a whole double emulsion unit. Reproduced from Clemente et al. (102).

Clemente et al. (102) explored the water/oil/water interface of phospholipid 1,2-dimyristoyl-*sn-glycerol*-3-phosphocholine [DMPC; 1% (wt/wt)] dissolved in a mixture of volatile solvents such as cyclohexane/chloroform (volume ratio 2:1) using microfluidic devices and investigated the role of μ-SAXS (a sketch of the device provided in Figure 7 Top). The structural characteristics of oil/DMPC bilayers were indicated by the decay in the intensity of the SAXS pattern with an inflection at *q* = 0.074 Å^–1^ corresponding to the first minimum value in the form factor of flat objects extending over large distances (Figure 7 Bottom left), which was also reported in previous studies involving the liquid/liquid (L/L) interface of w/o emulsions generated by a microfluidic apparatus (103). The bilayer thickness was found to shrink under treatment at 50°C for 1 h, but not to increase the order of the lipid bilayers, as suggested by the minimum position (*q* = 0.074 Å^–1^) shifting toward higher *q*-region (*q* = 0.088 Å ^–1^). The authors reported that the phase behavior and structural dynamics of phospholipids at the L/L interfaces can be detected well *via* micro-focusing SAXS (Figure 7 Bottom right), which may provide deeper insight into the role of double lipid emulsions in the food industry.

**FIGURE 8:**
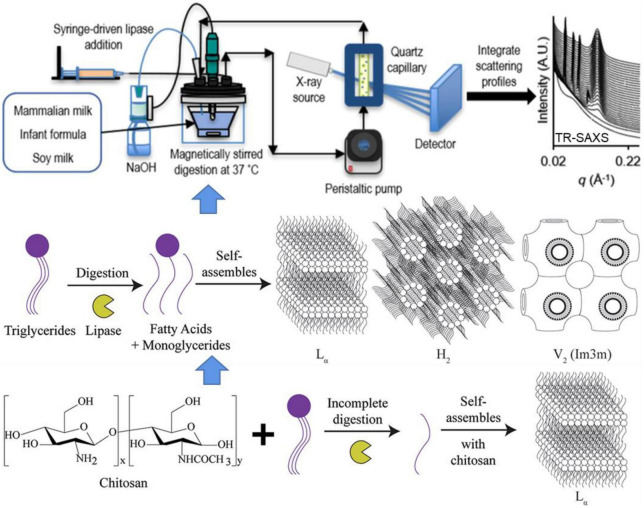
Schematic diagram showing the detection of self-assembled structures of lipids during the digestion of milk using a lipolysis device coupled with *in situ* TR-SAXS. Reprinted from Pham et al. (104).

Pham et al. (104) investigated the lipid self-assembly during *in vitro* digestion of bovine, human and goat milk using *in situ* TR-SAXS. The SAXS data revealed similar structural behavior during the early stages of the digestion of three types of milk, indicating lamellar (L_α_), inverse hexagonal (H_2_), and continuous cubic (V_2_) phases (Figure 8 Top and Middle). All the milk tested self-assembled into non-lamellar liquid crystalline structures, with coexisting lamellar phases associated with calcium soap formation. By tracking the changes in L_α_, H_2_, and V_2_ phases, the investigators concluded that different structures were formed during the digestion of all three infant formulas tested. During the digestion, soy and human milk that released long-chain fatty acids showed an inverse micellar cubic I_2_ phase at the oil–water interface, while bovine and goat milk yielded a greater proportion of medium-chain fatty acids tended to exhibit either the V_2_ or a H_2_ hexagonal phase. In addition, a TR-SAXS equipped with pH-stat or HHP system was used to monitor both the kinetics of lipolysis and structural behavior during the *in vitro* digestion of lipids in the presence of carbohydrates, such as chitosan (105), amylose (106), and other commercial supplements (as shown in Figure 8 Bottom).

Undoubtedly, the TR-SAXS (106–108) facilitates real-time monitoring of the crystallization of milk lipids under different treatments, but also assessment of the lipid self-assembly during *in vitro* digestion or under varying buffer conditions, which play a critical role in industrial applications involving the processing and storage of lipid-based foods.

### Other biomacromolecules

Traditionally, natural sausage casings are made from collagen-rich intestinal submucosa derived from bovine, porcine and ovine sources. Collagen arrangement in the intestinal submucosa provides strength to animal tissues, although the relationship between structure and strength is not well-characterized. Gunn et al. (109) demonstrated collagen fibril orientation, orientation index (OI) and *d*-spacing of bovine, porcine and ovine sausage casings using SAXS. The *d*-spacing was calculated in the range of 64.8–65.2 nm, and the collagen fibrils were arranged in planar layers with OI values of 0.86–0.91 based on the X-ray adsorption edge energies of all samples.

Pectin has been approved as Generally Recognized as Safe (GRAS) and as a food emulsifier, stabilizer, thickener, and gelling agent (110). Mendez et al. (111) elucidated the different emulsification mechanisms of three pectin sources (watermelon, citrus and apple) using SAXS. The two shoulder-like peaks in the low-*q* (*q* < ∼0.03 Å^–1^) and high-*q* regions were as attributed to intermolecular interactions and chain clusters, and the scattering of rod-like pectin chains, respectively. The *R*_*g*3_ value of all samples with cross-sections of rod-like pectin chains ranged from 1.4 to 2.3 nm. The P_2_ corresponding to the smallest structural level ranged from 1.8 to 2.8, suggesting the existence of flexible folded chains rather than ideal rigid rods. The *R*_*g2*_ values, referring to the size of the molecular clusters originating as a result of chain bending, determined for pectin solutions (20–38 nm) fall within the range previously determined by SAXS (6.3–42 nm) for pectin with different degrees of esterification.

Food-grade surfactants, like lauric arginate (LAE), have been used widely as a preservative against a wide range of food pathogens and spoilage organisms such as processed meats, dairy products and fruit juices (112). Nallamilli et al. (113) reported a coacervation complex of LAE with λ-carrageenan consisting of loosely packed and disordered LAE molecules with an internal bilayer-like structure indicated by a scattering intensity peak at *q* ≈ 0.161 Å^–1^, based on which, a periodicity value of *d* = 2π/*q*∼3.9 nm was calculated. This was consistent with the quantitative analysis involving fitting a Teubner-Strey structure factor yielding *d*-spacing of the lamellae from 3.75 to 4.01 nm. Formation of bilayer coacervates was observed at the LAE/carrageenan weight ratio of 2 and the maximum coacervation was detected at a ratio of 5.

## Conclusion and outlook

In the current review, recent advances in the structural characterization of food biomacromolecules using SAXS are summarized, including the principle, theoretical calculation methods, *in situ* capabilities, and applications. The unique feature of SAXS not only provides direct and rapid structural information of biomolecules in their native state but also facilitates the elucidation of conformational dynamics in real-time. SAXS combined with online chromatography represents a fascinating tool for separating and detecting mixtures and flexible systems synchronously, such as disordered fragments in proteins, long-chain ribonucleic acid and IDPs, which significantly widens the range of SAXS applications. Microfluidics installed in SAXS facilitate the study of binding kinetics by reducing the required sample volume to the sub-microliter level. A combination of T-jump or P-jump pump coupled to TR-SAXS allows direct tracking of the structural dynamics triggered by changes in temperature or pressure over a set time period ranging from microseconds to hours. Based on the volume of studies reported, SAXS is becoming a promising tool for monitoring the structure, conformation, interaction, kinetics, and reaction of biomacromolecules to provide molecular insights into the structure-function relationship of biomolecules in different food processing applications.

In the next decade, the application of the fourth-generation of high-brilliance synchrotron facilities will provide insight into the biomacromolecules at the atomic and molecular levels, and promote cutting-edge research *via* high-resolution imaging, ultrafast process exploration and advanced structural analysis based on SAXS. Furthermore, with the development of artificial intelligence (AI), research that was previously inconceivable or wildly impractical, especially involving protein structure prediction, is now feasible. We have reason to anticipate potential future applications of AI coupled with high-brilliance SAXS in understanding not merely the individual biomacromolecules and complexes in the food industry, but entire cells or even tissues in life science.

## Author contributions

YS: conceptualization, methodology, software, writing—review and editing, revise, and supervision. XL and RC: software and writing—review and editing. FL: writing and editing. SW: writing—review and editing, revise, and supervision.

## References

[1] Okolie CL Akanbi TO Mason B Udenigwe CC Aryee ANA. Influence of conventional and recent extraction technologies on physicochemical properties of bioactive macromolecules from natural sources: a review. *Food Res Int.* (2019) 116:827–39. 10.1016/j.foodres.2018.09.018 30717014

[2] Clore GM Iwahara J. Theory, practice, and applications of paramagnetic relaxation enhancement for the characterization of transient low-population states of biological macromolecules and their complexes. *Chem Rev.* (2009) 109:4108–39. 10.1021/cr900033p 19522502 PMC2825090

[3] Trampari S Neumann C Hjorth-Jensen SJ Shahsavar A Quistgaard EM Nissen P. Insights into the mechanism of high lipid-detergent crystallization of membrane proteins. *J Appl Crystallogr.* (2021) 54:1775–83. 10.1073/pnas.0606149103 17050688 PMC1616942

[4] Wehbie M Onyia KK Mahler F Le Roy A Deletraz A Bouchemal I Maltose-based fluorinated surfactants for membrane-protein extraction and stabilization. *Langmuir.* (2021) 37:2111–22. 10.1021/acs.langmuir.0c03214 33539092

[5] Svergun DI Koch MHJ. Small-angle scattering studies of biological macromolecules in solution. *Rep Prog Phys.* (2003) 66:1735–82.

[6] Gräwert TW Svergun DI. Structural modeling using solution small-angle X-ray scattering (SAXS). *J Mol Biol.* (2020) 432:3078–92. 10.1016/j.jmb.2020.01.030 32035901

[7] de Kruif CG. The structure of casein micelles: a review of small-angle scattering data. *J Appl Crystallogr.* (2014) 47:1479–89.

[8] de Kruif CG Huppertz T Urban VS Petukhov AV. Casein micelles and their internal structure. *Adv Colloid Interface Sci.* (2012) 171:36–52. 22381008 10.1016/j.cis.2012.01.002

[9] Huang J Wang Z Fan L Ma S. A review of wheat starch analyses: methods, techniques, structure and function. *Int J Biol Macromol.* (2022) 203:130–42. 35093434 10.1016/j.ijbiomac.2022.01.149

[10] Lopez-Rubio A Hernandez-Munoz P Catala R Gavara R Lagaron JM. Improving packaged food quality and safety. Part 1: synchrotron X-ray analysis. *Food Addit Contam.* (2005) 22:988–93. 10.1080/02652030500246370 16227183

[11] Rostamabadi H Falsafi SR Assadpour E Jafari SM. Evaluating the structural properties of bioactive-loaded nanocarriers with modern analytical tools. *Compr Rev Food Sci Food Saf.* (2020) 19:3266–322. 10.1111/1541-4337.12653 33337066

[12] Smith G Brok E Jensen GV Midtgaard SR Skar-Gislinge N Arleth L. Casein studied by X-ray and neutron scattering. *Abstracts of Papers of the American Chemical Society.* Vol. 256. Washington, DC: American Chemical Society (2018).

[13] Smith GN Brok E Christiansen MV Ahrne L. Casein micelles in milk as sticky spheres. *Soft Matter.* (2020) 16:9955–63. 33034319 10.1039/d0sm01327g

[14] Gilbert EP. Small-angle X-Ray and neutron scattering in food colloids. *Curr Opin Colloid Interface Sci.* (2019) 42:55–72.

[15] Schneidman-Duhovny D Hammel M Sali A. Macromolecular docking restrained by a small angle X-ray scattering profile. *J Struct Biol.* (2011) 173:461–71. 10.1016/j.jsb.2010.09.023 20920583 PMC3040266

[16] Singh AK. Chapter 4 - Experimental methodologies for the characterization of nanoparticles. In: Singh AK editor. *Engineered Nanoparticles.* Boston, MA: Academic Press (2016). p. 125–70.

[17] Molodenskiy DS Svergun DI Kikhney AG. Artificial neural networks for solution scattering data analysis. *Structure.* (2022) 30:900–8.e2. 35413244 10.1016/j.str.2022.03.011

[18] Guinier A. La diffraction des rayons X aux très petits angles : application à l’étude de phénomènes ultramicroscopiques. *Ann Phys.* (1939) 11:161–237.

[19] Glatter O. A new method for the evaluation of small-angle scattering data. *J. Appl Crystallogr.* (1977) 10:415–21.

[20] Svergun DI. Determination of the regularization parameter in indirect-transform methods using perceptual criteria. *J Appl Crystallogr.* (1992) 25:495–503.

[21] Konarev PV Volkov VV Sokolova AV Koch MHJ Svergun DI. PRIMUS: a windows PC-based system for small-angle scattering data analysis. *J Appl Crystallogr.* (2003) 36:1277–82.

[22] Hopkins JB Gillilan RE Skou S. BioXTAS RAW: improvements to a free open-source program for small-angle X-ray scattering data reduction and analysis. *J Appl Crystallogr.* (2017) 50:1545–53. 10.1107/S1600576717011438 29021737 PMC5627684

[23] Hansen S. Bayesian estimation of hyperparameters for indirect Fourier transformation in small-angle scattering. *J Appl Crystallogr.* (2000) 33:1415–21.

[24] Porod G. Die Röntgenkleinwinkelstreuung von dichtgepackten kolloiden Systemen. *Kolloid Zeitschrift.* (1952) 125:51–7.

[25] Trewhella J. Small angle scattering and structural biology: data quality and model validation. In: Nakamura H Kleywegt G Burley SK Markley JL editors. *Integrative Structural Biology with Hybrid Methods.* Berlin: Springer (2018). p. 77–100. 10.1007/978-981-13-2200-6_7 30617825

[26] Pilz I Glatter O Kratky O. Small-angle X-ray scattering. *Methods Enzymol.* (1979) 61:148–249. 481226 10.1016/0076-6879(79)61013-3

[27] Bizien T Durand D Roblina P Thureau A Vachette P Pérez J. A brief survey of state-of-the-art BioSAXS. *Protein Pept Lett.* (2016) 23:217–31. 10.2174/0929866523666160106153655 26732245

[28] Rambo RP Tainer JA. Characterizing flexible and intrinsically unstructured biological macromolecules by SAS using the porod-debye law. *Biopolymers.* (2011) 95:559–71. 10.1002/bip.21638 21509745 PMC3103662

[29] Sun Y Tai Z Yan T Dai Y Hemar Y Li N. Unveiling the structure of the primary caseinate particle using small-angle X-ray scattering and simulation methodologies. *Food Res Int.* (2021) 149:110653. 10.1016/j.foodres.2021.110653 34600655

[30] Wang G Xu L-F Shen J-L Yao G-B Ge Z-L Li W-Q Iterative and accurate determination of small angle X-ray scattering background. *Nucl Sci Tech.* (2016) 27:105.

[31] Svergun DI. Restoring low resolution structure of biological macromolecules from solution scattering using simulated annealing. *Biophys J.* (1999) 76:2879–86. 10.1016/S0006-3495(99)77443-6 10354416 PMC1300260

[32] Svergun DI Petoukhov MV Koch MHJ. Determination of domain structure of proteins from X-ray solution scattering. *Biophys J.* (2001) 80:2946–53. 10.1016/S0006-3495(01)76260-1 11371467 PMC1301478

[33] Grant TD. Ab initio electron density determination directly from solution scattering data. *Nat Methods.* (2018) 15:191–3. 29377013 10.1038/nmeth.4581

[34] Franke D Jeffries CM Svergun DI. Machine learning methods for X-ray scattering data analysis from biomacromolecular solutions. *Biophys J.* (2018) 114:2485–92. 10.1016/j.bpj.2018.04.018 29874600 PMC6129182

[35] He H Liu C Liu H. Model reconstruction from small-angle X-ray scattering data using deep learning methods. *iScience.* (2020) 23:100906. 10.1016/j.isci.2020.100906 32092702 PMC7037568

[36] Hura GL Hodge CD Rosenberg D Guzenko D Duarte JM Monastyrskyy B Small angle X-ray scattering-assisted protein structure prediction in CASP13 and emergence of solution structure differences. *Proteins.* (2019) 87:1298–314. 10.1002/prot.25827 31589784 PMC6851496

[37] Panjkovich A Svergun DI. Deciphering conformational transitions of proteins by small angle X-ray scattering and normal mode analysis. *Phys Chem Chem Phys.* (2016) 18:5707–19. 10.1039/c5cp04540a 26611321

[38] Petoukhov MV Svergun DI. Global rigid body modeling of macromolecular complexes against small-angle scattering data. *Biophys J.* (2005) 89:1237–50. 10.1529/biophysj.105.064154 15923225 PMC1366608

[39] Petoukhov MV Franke D Shkumatov AV Tria G Kikhney AG Gajda M New developments in the ATSAS program package for small-angle scattering data analysis. *J Appl Crystallogr.* (2012) 45:342–50. 25484842 10.1107/S0021889812007662 PMC4233345

[40] Hou J Adhikari B Tanner JJ Cheng J. SAXSDom: modeling multidomain protein structures using small-angle X-ray scattering data. *Proteins.* (2020) 88:775–87. 10.1002/prot.25865 31860156 PMC7230021

[41] Svergun D Barberato C Koch MHJ. CRYSOL– a program to evaluate X-ray solution scattering of biological macromolecules from atomic coordinates. *J Appl Crystallogr.* (1995) 28:768–73.

[42] Kozin MB Svergun DI. Automated matching of high- and low-resolution structural models. *J Appl Crystallogr.* (2001) 34:33–41.

[43] Tria G Mertens HDT Kachala M Svergun DI. Advanced ensemble modelling of flexible macromolecules using X-ray solution scattering. *IUCrJ.* (2015) 2:207–17. 10.1107/S205225251500202X 25866658 PMC4392415

[44] Konarev PV Svergun DI. Direct shape determination of intermediates in evolving macromolecular solutions from small-angle scattering data. *IUCrJ.* (2018) 5:402–9. 10.1107/S2052252518005900 30002841 PMC6038953

[45] Hermann MR Hub JS. SAXS-restrained ensemble simulations of intrinsically disordered proteins with commitment to the principle of maximum entropy. *J Chem Theory Comput.* (2019) 15:5103–15. 10.1021/acs.jctc.9b00338 31402649

[46] Lincoff J Haghighatlari M Krzeminski M Teixeira JMC Gomes G-NW Gradinaru CC Extended experimental inferential structure determination method in determining the structural ensembles of disordered protein states. *Commun Chem.* (2020) 3:74. 10.1038/s42004-020-0323-0 32775701 PMC7409953

[47] Bottaro S Bengtsen T Lindorff-Larsen K. Integrating molecular simulation and experimental data: a bayesian/maximum entropy reweighting approach. In: Gáspári Z editor. *Structural Bioinformatics: Methods and Protocols.* New York, NY: Springer US (2020). p. 219–40. 10.1007/978-1-0716-0270-6_15 32006288

[48] Konarev PV Gruzinov AY Mertens HDT Svergun DI. Restoring structural parameters of lipid mixtures from small-angle X-ray scattering data. *J Appl Crystallogr.* (2021) 54:169–79. 10.1107/S1600576720015368 33833646 PMC7941313

[49] Konarev PV Petoukhov MV Dadinova LA Fedorova NV Volynsky PE Svergun DI BILMIX: a new approach to restore the size polydispersity and electron density profiles of lipid bilayers from liposomes using small-angle X-ray scattering data. *J Appl Crystallogr.* (2020) 53:236–43.

[50] Petukhov MV Konarev PV Dadinova LA Fedorova NV Volynsky PE Svergun DI Quasi-atomistic approach to modeling of liposomes. *Crystallogr Rep.* (2020) 65:258–63.

[51] Manalastas-Cantos K Konarev PV Hajizadeh NR Kikhney AG Petoukhov MV Molodenskiy DS ATSAS 3.0: expanded functionality and new tools for small-angle scattering data analysis. *J Appl Crystallogr.* (2021) 54:343–55. 10.1107/S1600576720013412 33833657 PMC7941305

[52] Schneidman-Duhovny D Hammel M Tainer JA Sali A. FoXS, FoXSDock and MultiFoXS: single-state and multi-state structural modeling of proteins and their complexes based on SAXS profiles. *Nucleic Acids Res.* (2016) 44:W424–9. 10.1093/nar/gkw389 27151198 PMC4987932

[53] Karaca E Bonvin AMJJ. On the usefulness of ion-mobility mass spectrometry and SAXS data in scoring docking decoys. *Acta Crystallogr D Biol Crystallogr.* (2013) 69:683–94. 10.1107/S0907444913007063 23633577

[54] Ignatov M Kazennov A Kozakov D. ClusPro FMFT-SAXS: ultra-fast filtering using small-angle X-ray scattering data in protein docking. *J Mol Biol.* (2018) 430:2249–55. 10.1016/j.jmb.2018.03.010 29626538

[55] Jiménez-García B Bernadó P Fernández-Recio J. Structural characterization of protein–protein interactions with pyDockSAXS. In: Gáspári Z editor. *Structural Bioinformatics: Methods and Protocols.* New York, NY: Springer US (2020). p. 131–44. 10.1007/978-1-0716-0270-6_10 32006283

[56] Sønderby P Rinnan Å Madsen JJ Harris P Bukrinski JT Peters GHJ. Small-angle X-ray scattering data in combination with Rosettadock improves the docking energy landscape. *J Chem Inf Model.* (2017) 57:2463–75. 10.1021/acs.jcim.6b00789 28853875

[57] Schneidman-Duhovny D Hammel M. Modeling structure and dynamics of protein complexes with SAXS profiles. *Methods Mol Biol.* (2018) 1764:449–73. 29605933 10.1007/978-1-4939-7759-8_29 PMC6022765

[58] Schindler Christina EM de Vries SJ Sasse A Zacharias M. SAXS data alone can generate high-quality models of protein-protein complexes. *Structure.* (2016) 24:1387–97. 10.1016/j.str.2016.06.007 27427479

[59] Huang W Ravikumar KM Parisien M Yang S. Theoretical modeling of multiprotein complexes by iSPOT: integration of small-angle X-ray scattering, hydroxyl radical footprinting, and computational docking. *J Struct Biol.* (2016) 196:340–9. 10.1016/j.jsb.2016.08.001 27496803 PMC5118146

[60] Molodenskiy DS Svergun DI Mertens HDT. MPBuilder: a PyMOL plugin for building and refinement of solubilized membrane proteins against small angle X-ray scattering data. *J Mol Biol.* (2021) 433:166888. 10.1016/j.jmb.2021.166888 33631193 PMC8135126

[61] Mahieu E Gabel F. Biological small-angle neutron scattering: recent results and development. *Acta Crystallogr D Struct Biol.* (2018) 74:715–26. 30082507 10.1107/S2059798318005016

[62] Brennich M Hutin S Weinhaupl K Schanda P Pernot P. Beyond size exclusion: online liquid chromatography for BioSAXS. *Acta Crystallogr A Found Adv.* (2017) 73:A83–83.

[63] Panjkovich A Svergun DI. CHROMIXS: automatic and interactive analysis of chromatography-coupled small-angle X-ray scattering data. *Bioinformatics.* (2018) 34:1944–6. 10.1093/bioinformatics/btx846 29300836 PMC5972624

[64] Shkumatov AV Strelkov SV. DATASW, a tool for HPLC–SAXS data analysis. *Acta Crystallogr D Biol Crystallogr.* (2015) 71:1347–50. 10.1107/S1399004715007154 26057674

[65] Malaby AW Chakravarthy S Irving TC Kathuria SV Bilsel O Lambright DG. Methods for analysis of size-exclusion chromatography–small-angle X-ray scattering and reconstruction of protein scattering. *J Appl Crystallogr.* (2015) 48:1102–13. 10.1107/S1600576715010420 26306089 PMC4520288

[66] Konarev PV Graewert MA Jeffries CM Fukuda M Cheremnykh TA Volkov VV EFAMIX, a tool to decompose inline chromatography SAXS data from partially overlapping components. *Protein Sci.* (2022) 31:269–82. 10.1002/pro.4237 34767272 PMC8740826

[67] Brookes E Vachette P Rocco M Perez J. US-SOMO HPLC-SAXS module: dealing with capillary fouling and extraction of pure component patterns from poorly resolved SEC-SAXS data. *J Appl Crystallogr.* (2016) 49:1827–41. 10.1107/S1600576716011201 27738419 PMC5045733

[68] Clulow AJ Salim M Hawley A Boyd BJ. A closer look at the behaviour of milk lipids during digestion. *Chem Phys Lipids.* (2018) 211:107–16. 10.1016/j.chemphyslip.2017.10.009 29100945

[69] Ravishankar H Nors Pedersen M Sitsel A Li C Duelli A Levantino M Tracking Ca2+ATPase intermediates in real-time by X-ray solution scattering. *Sci Adv.* (2020) 6:eaaz0981. 10.1126/sciadv.aaz0981 32219166 PMC7083613

[70] Heyes DJ Hardman SJO Pedersen MN Woodhouse J De La Mora E Wulff M Light-induced structural changes in a full-length cyanobacterial phytochrome probed by time-resolved X-ray scattering. *Commun Biol.* (2019) 2:1. 10.1038/s42003-018-0242-0 30740537 PMC6318211

[71] Thompson MC Barad BA Wolff AM Sun Cho H Schotte F Schwarz DMC Temperature-jump solution X-ray scattering reveals distinct motions in a dynamic enzyme. *Nat Chem.* (2019) 11:1058–66. 10.1038/s41557-019-0329-3 31527847 PMC6815256

[72] Rimmerman D Leshchev D Hsu DJ Hong J Abraham B Henning R Revealing fast structural dynamics in ph-responsive peptides with time-resolved X-ray scattering. *J Phys Chem B.* (2019) 123:2016–21. 10.1021/acs.jpcb.9b00072 30763085 PMC6533112

[73] Kim TW Lee SJ Jo J Kim JG Ki H Kim CW Protein folding from heterogeneous unfolded state revealed by time-resolved X-ray solution scattering. *Proc Natl Acad Sci USA.* (2020) 117:14996–5005. 10.1073/pnas.1913442117 32541047 PMC7334511

[74] Hsu DJ Leshchev D Kosheleva I Kohlstedt KL Chen LX. Unfolding bovine α-lactalbumin with T-jump: characterizing disordered intermediates via time-resolved x-ray solution scattering and molecular dynamics simulations. *J Chem Phys.* (2021) 154:105101. 10.1063/5.0039194 33722011 PMC7943248

[75] Kuang Q Xu J Liang Y Xie F Tian F Zhou S Lamellar structure change of waxy corn starch during gelatinization by time-resolved synchrotron SAXS. *Food Hydrocoll.* (2017) 62:43–8.

[76] Hempt C Gontsarik M Buerki-Thurnherr T Hirsch C Salentinig S. Nanostructure generation during milk digestion in presence of a cell culture model simulating the small intestine. *J Colloid Interface Sci.* (2020) 574:430–40. 10.1016/j.jcis.2020.04.059 32344233

[77] Krishnamoorthy K Kewalramani S Ehlen A Moreau LM Mirkin CA de la Cruz MO Enzymatic degradation of DNA probed by in situ X-ray scattering. *ACS Nano.* (2019) 13:11382–91. 31513370 10.1021/acsnano.9b04752

[78] Narayanan T Konovalov O. Synchrotron scattering methods for nanomaterials and soft matter research. *Materials.* (2020) 13:752. 10.3390/ma13030752 PMC7040635 32041363

[79] Ilhan-Ayisigi E Yaldiz B Bor G Yaghmur A Yesil-Celiktas O. Advances in microfluidic synthesis and coupling with synchrotron SAXS for continuous production and real-time structural characterization of nano-self-assemblies. *Colloids Surf B Biointerfaces.* (2021) 201:111633. 10.1016/j.colsurfb.2021.111633 33639513

[80] Berntsson O Terry AE Plivelic TS. A setup for millisecond time-resolved X-ray solution scattering experiments at the CoSAXS beamline at the MAX IV Laboratory. *J Synchrotron Radiat.* (2022) 29:555–62. 10.1107/S1600577522000996 35254321 PMC8900842

[81] Balasubramaniam VM. Process development of high pressure-based technologies for food: research advances and future perspectives. *Curr Opin Food Sci.* (2021) 42:270–277.

[82] Ferreira Zielinski AA Sanchez-Camargo ADP Benvenutti L Ferro DM Dias JL Salvador Ferreira SR. High-pressure fluid technologies: recent approaches to the production of natural pigments for food and pharmaceutical applications. *Trends Food Sci Technol.* (2021) 118:850–69.

[83] Harish B Gillilan RE Zou J Wang J Raleigh DP Royer CA. Protein unfolded states populated at high and ambient pressure are similarly compact. *Biophys J.* (2021) 120:2592–8. 10.1016/j.bpj.2021.04.031 33961866 PMC8390852

[84] Yang S Tyler AII Ahrné L Kirkensgaard JJK. Skimmed milk structural dynamics during high hydrostatic pressure processing from in situ SAXS. *Food Res Int.* (2021) 147:110527. 10.1016/j.foodres.2021.110527 34399505

[85] Rai DK Gillilan RE Huang Q Miller R Ting E Lazarev A High-pressure small-angle X-ray scattering cell for biological solutions and soft materials. *J Appl Crystallogr.* (2021) 54:111–22. 10.1107/S1600576720014752 33841059 PMC7941318

[86] Vella J Hemar Y Gu Q Wu ZR Li N Söhnel T. In-situ SAXS investigation of high-pressure triglyceride polymorphism in milk cream and anhydrous milk fat. *LWT.* (2021) 135:110174.

[87] Lehmkühler F Schroer MA Markmann V Frenzel L Möller J Lange H Kinetics of pressure-induced nanocrystal superlattice formation. *Phys Chem Chem Phys.* (2019) 21:21349–54. 10.1039/c9cp04658e 31531471

[88] Loveday SM. Food proteins: technological, nutritional, and sustainability attributes of traditional and emerging proteins. *Annu Rev Food Sci Technol.* (2019) 10:311–39. 10.1146/annurev-food-032818-121128 30649962

[89] Alrosan M Tan TC Easa AM Gammoh S Alu’datt MH. Molecular forces governing protein-protein interaction: structure-function relationship of complexes protein in the food industry. *Crit Rev Food Sci Nutr.* (2022) 62:4036–52. 10.1080/10408398.2021.1871589 33455424

[90] Yang Z de Campo L Gilbert EP Knott R Cheng L Storer B Effect of NaCl and CaCl_2_ concentration on the rheological and structural characteristics of thermally-induced quinoa protein gels. *Food Hydrocoll.* (2022) 124:107350.

[91] Pohl C Mahapatra S Kulakova A Streicher W Peters GHJ Nørgaard A Combination of high throughput and structural screening to assess protein stability – A screening perspective. *Eur J Pharm Biopharm.* (2022) 171:1–10. 10.1016/j.ejpb.2021.08.018 34826593

[92] Yang Z Gu Q Banjar W Li N Hemar Y. In situ study of skim milk structure changes under high hydrostatic pressure using synchrotron SAXS. *Food Hydrocoll.* (2018) 77:772–6.

[93] Comerford KB Papanikolaou Y Jones JM Rodriguez J Slavin J Angadi S Toward an evidence-based definition and classification of carbohydrate food quality: an expert panel report. *Nutrients.* (2021) 13:2667. 10.3390/nu13082667 34444826 PMC8398407

[94] Zeng X Zheng B Li T Chen L. How to synchronously slow down starch digestion and retrogradation: a structural analysis study. *Int J Biol Macromol.* (2022) 212:43–53. 10.1016/j.ijbiomac.2022.05.099 35597377

[95] Xu J Blennow A Li X Chen L Liu X. Gelatinization dynamics of starch in dependence of its lamellar structure, crystalline polymorphs and amylose content. *Carbohydr Polym.* (2020) 229:115481. 10.1016/j.carbpol.2019.115481 31826407

[96] Xu J Li Z Zhong Y Zhou Q Lv Q Chen L The effects of molecular fine structure on rice starch granule gelatinization dynamics as investigated by in situ small-angle X-ray scattering. *Food Hydrocoll.* (2021) 121:107014.

[97] Díaz-Calderón P Simone E Tyler AII Enrione J Foster T. A structural study of the self-association of different starches in presence of bacterial cellulose fibrils. *Carbohydr Polym.* (2022) 288:119361. 10.1016/j.carbpol.2022.119361 35450626

[98] Li C Gong B Hu Y Liu X Guan X Zhang B. Combined crystalline, lamellar and granular structural insights into in vitro digestion rate of native starches. *Food Hydrocoll.* (2020) 105:105823.

[99] Yang Z Swedlund P Hemar Y Mo G Wei Y Li Z Effect of high hydrostatic pressure on the supramolecular structure of corn starch with different amylose contents. *Int J Biol Macromol.* (2016) 85:604–14. 26778159 10.1016/j.ijbiomac.2016.01.018

[100] Bascuas S Morell P Hernando I Quiles A. Recent trends in oil structuring using hydrocolloids. *Food Hydrocoll.* (2021) 118:106612.

[101] Wang G Chen H Wang L Zou Y Wan Z Yang X. Formation of protein oleogels via capillary attraction of engineered protein particles. *Food Hydrocoll.* (2022) 133:107912.

[102] Clemente I Torbensen K Di Cola E Rossi F Ristori S Abou-Hassan A. Exploring the water/oil/water interface of phospholipid stabilized double emulsions by micro-focusing synchrotron SAXS. *RSC Adv.* (2019) 9:33429–35. 10.1039/c9ra05894j 35529139 PMC9073385

[103] Di Cola E Torbensen K Clemente I Rossi F Ristori S Abou-Hassan A. Lipid-stabilized water–oil interfaces studied by microfocusing small-angle X-ray scattering. *Langmuir.* (2017) 33:9100–5. 10.1021/acs.langmuir.7b02076 28816051

[104] Pham AC Peng K-Y Salim M Ramirez G Hawley A Clulow AJ Correlating digestion-driven self-assembly in milk and infant formulas with changes in lipid composition. *ACS Appl Bio Mater.* (2020) 3:3087–98. 10.1021/acsabm.0c00131 32455340 PMC7241073

[105] May KL Tangso KJ Hawley A Boyd BJ Clulow AJ. Interaction of chitosan-based dietary supplements with fats during lipid digestion. *Food Hydrocoll.* (2020) 108:105965.

[106] Jia X Sun S Chen B Zheng B Guo Z. Understanding the crystal structure of lotus seed amylose–long-chain fatty acid complexes prepared by high hydrostatic pressure. *Food Res Int.* (2018) 111:334–41. 10.1016/j.foodres.2018.05.053 30007694

[107] Dyett B Zychowski L Bao L Meikle TG Peng S Yu H Crystallization of Femtoliter Surface Droplet Arrays Revealed by Synchrotron Small-Angle X-ray Scattering. *Langmuir.* (2018) 34:9470–6. 10.1021/acs.langmuir.8b01252 30021434

[108] Yaghmur A Lotfi S Ariabod SA Bor G Gontsarik M Salentinig S. Internal lamellar and inverse hexagonal liquid crystalline phases during the digestion of krill and astaxanthin oil-in-water emulsions. *Front Bioeng Biotechnol.* (2019) 7:384. 10.3389/fbioe.2019.00384 31867316 PMC6906996

[109] Gunn S Sizeland KH Wells HC Haverkamp RG. Collagen arrangement and strength in sausage casings produced from natural intestines. *Food Hydrocoll.* (2022) 129:107612.

[110] Li Z Xiong Y Wang Y Zhang Y Luo Y. Low density lipoprotein-pectin complexes stabilized high internal phase pickering emulsions: the effects of pH conditions and mass ratios. *Food Hydrocoll.* (2023) 134:108004.

[111] Mendez DA Fabra MJ Martínez-Abad A Martínez-Sanz M Gorria M López-Rubio A. Understanding the different emulsification mechanisms of pectin: comparison between watermelon rind and two commercial pectin sources. *Food Hydrocoll.* (2021) 120:106957.

[112] Hu X Huang E Barringer SA Yousef AE. Factors affecting *Alicyclobacillus acidoterrestris* growth and guaiacol production and controlling apple juice spoilage by lauric arginate and ϵ-polylysine. *LWT.* (2020) 119:108883.

[113] Nallamilli T Ketomaeki M Prozeller D Mars J Morsbach S Mezger M Complex coacervation of food grade antimicrobial lauric arginate with lambda carrageenan. *Curr Res Food Sci.* (2021) 4:53–62. 10.1016/j.crfs.2021.01.003 33665619 PMC7902899

[114] Jiménez-García B Pons C Svergun DI Bernadó P Fernández-Recio J. pyDockSAXS: protein–protein complex structure by SAXS and computational docking. *Nucleic Acids Res.* (2015) 43:W356–61. 25897115 10.1093/nar/gkv368 PMC4489248

[115] Xia B Vajda S Kozakov D. Accounting for pairwise distance restraints in FFT-based protein–protein docking. *Bioinformatics.* (2016) 32:3342–4. 10.1093/bioinformatics/btw306 27357172 PMC6095118

[116] Chen P-C Masiewicz P Rybin V Svergun D Hennig J. A General Small-Angle X-ray Scattering-Based Screening Protocol Validated for Protein–RNA Interactions. *ACS Comb Sci.* (2018) 20:197–202. 10.1021/acscombsci.8b00007 29553252

[117] Graewert M Vela SD Grwert TW Molodenskiy DS Jeffries CMJC. Adding size exclusion chromatography (SEC) and light scattering (LS) devices to obtain high-quality small angle X-ray scattering (SAXS) data. *Crystals.* (2020) 10:975.

[118] Graewert MA Franke D Jeffries CM Blanchet CE Ruskule D Kuhle K Automated pipeline for purification, biophysical and X-ray analysis of biomacromolecular solutions. *Sci Rep.* (2015) 5:10734. 10.1038/srep10734 26030009 PMC5377070

[119] Takemasa M Yuguchi Y Kitamura S. Size and shape of cycloamylose estimated using column chromatography coupled with small-angle X-ray scattering. *Food Hydrocoll.* (2020) 108:105948.

[120] Watanabe Y. Size-exclusion chromatography combined with solution X-ray scattering measurement of the heat-induced aggregates of water-soluble proteins at low ionic strength in a neutral solution. *J Chromatogr A.* (2019) 1603:190–8. 10.1016/j.chroma.2019.06.042 31277950

[121] Watanabe Y Inoko Y. Characterization of a large glycoprotein proteoglycan by size-exclusion chromatography combined with light and X-ray scattering methods. *J Chromatogr A.* (2013) 1303:100–4. 10.1016/j.chroma.2013.06.048 23859798

[122] Ryan TM Trewhella J Murphy JM Keown JR Casey L Pearce FG An optimized SEC-SAXS system enabling high X-ray dose for rapid SAXS assessment with correlated UV measurements for biomolecular structure analysis. *J Appl Crystallogr.* (2018) 51:97–111.

[123] Kim JH Min B Yun YD Choi HJ Jin KS. Size-exclusion chromatography coupled with small-angle X-ray scattering on the 4C small-angle X-ray scattering beamline at pohang light source II. *Bull Korean Chem Soc.* (2020) 41:1052–5.

[124] Inoue R Nakagawa T Morishima K Sato N Okuda A Urade R Newly developed Laboratory-based Size exclusion chromatography Small-angle x-ray scattering System (La-SSS). *Sci Rep.* (2019) 9:12610. 10.1038/s41598-019-48911-w 31471544 PMC6717197

[125] Bucciarelli S Midtgaard SR Nors Pedersen M Skou S Arleth L Vestergaard B. Size-exclusion chromatography small-angle X-ray scattering of water soluble proteins on a laboratory instrument. *J Appl Crystallogr.* (2018) 51:1623–32. 10.1107/S1600576718014462 30546289 PMC6276278

[126] Banc A Pincemaille J Costanzo S Chauveau E Appavou M-S Morel M-H Phase separation dynamics of gluten protein mixtures. *Soft Matter.* (2019) 15:6160–70. 31317157 10.1039/c9sm00966c

[127] Chen D Kuzmenko I Ilavsky J Pinho L Campanella O. Structural evolution during gelation of pea and whey proteins envisaged by time-resolved ultra-small-angle x-ray scattering (USAXS). *Food Hydrocoll.* (2022) 126:107449.

[128] Chen D Zhu X Ilavsky J Whitmer T Hatzakis E Jones OG Polyphenols Weaken Pea Protein Gel by Formation of Large Aggregates with Diminished Noncovalent Interactions. *Biomacromolecules.* (2021) 22:1001–14. 10.1021/acs.biomac.0c01753 33494594

[129] Tsung K-L Ilavsky J Padua GW. Formation and characterization of zein-based oleogels. *J Agric Food Chem.* (2020) 68:13276–81. 10.1021/acs.jafc.0c00184 33047955

[130] Da Vela S Braun MK Dörr A Greco A Möller J Fu Z Kinetics of liquid–liquid phase separation in protein solutions exhibiting LCST phase behavior studied by time-resolved USAXS and VSANS. *Soft Matter.* (2016) 12:9334–41. 10.1039/c6sm01837h 27830221

[131] Mao Y Su Y Hsiao BS. Probing structure and orientation in polymers using synchrotron small- and wide-angle X-ray scattering techniques. *Eur Polym J.* (2016) 81:433–46.

[132] Yamamoto K Suzuki S Kitamura S Yuguchi Y. Gelation and structural formation of amylose by in situ neutralization as observed by small-angle X-ray scattering. *Gels.* (2018) 4:57. 10.3390/gels4030057 PMC6209274 30674833

[133] Royes J Bjørnestad VA Brun G Narayanan T Lund R Tribet C. Transition kinetics of mixed lipid:photosurfactant assemblies studied by time-resolved small angle X-ray scattering. *J Colloid Interface Sci.* (2022) 610:830–41. 10.1016/j.jcis.2021.11.133 34887060

[134] Gontsarik M Yaghmur A Salentinig S. Dispersed liquid crystals as pH-adjustable antimicrobial peptide nanocarriers. *J Colloid Interface Sci.* (2021) 583:672–82. 10.1016/j.jcis.2020.09.081 33039864

[135] Yaghmur A Rappolt M Jonassen ALU Schmitt M Larsen SW. In situ monitoring of the formation of lipidic non-lamellar liquid crystalline depot formulations in synovial fluid. *J Colloid Interface Sci.* (2021) 582:773–81. 32916575 10.1016/j.jcis.2020.08.084

[136] Komorowski K Schaeper J Sztucki M Sharpnack L Brehm G Köster S Vesicle adhesion in the electrostatic strong-coupling regime studied by time-resolved small-angle X-ray scattering. *Soft Matter.* (2020) 16:4142–54. 32319505 10.1039/d0sm00259c

[137] Khaliqi K Ghazal A Azmi IDM Amenitsch H Mortensen K Salentinig S Direct monitoring of lipid transfer on exposure of citrem nanoparticles to an ethanol solution containing soybean phospholipids by combining synchrotron SAXS with microfluidics. *Analyst.* (2017) 142:3118–26. 10.1039/c7an00860k 28744529

